# Fast and Green Method to Control Frauds of Geographical Origin in Traded Cuttlefish Using a Portable Infrared Reflective Instrument

**DOI:** 10.3390/foods10081678

**Published:** 2021-07-21

**Authors:** Sarah Currò, Stefania Balzan, Lorenzo Serva, Luciano Boffo, Jacopo Carlo Ferlito, Enrico Novelli, Luca Fasolato

**Affiliations:** 1 Department of Comparative Biomedicine and Food Science, University of Padova, Agripolis, Viale dell’Università 16, 35020 Legnaro, Italy; sarah.curro@phd.unipd.it (S.C.); enrico.novelli@unipd.it (E.N.); luca.fasolato@unipd.it (L.F.); 2 Department of Animal Medicine, Production and Health, University of Padova, Agripolis, Viale dell’Università 16, 35020 Legnaro, Italy; lorenzo.serva@unipd.it; 3 ITPhotonics S.r.l., Via Astico, 39, 36030 Fara Vicentino, Italy; luc.boffo@gmail.com; 4 BluPesca S.r.l., Isola Saloni, 59, 30015 Chioggia, Italy; j.ferlito@itphotonics.com

**Keywords:** traceability, authenticity, machine learning, NIRS, cephalopods, mislabeling

## Abstract

An appropriate seafood origin identification is essential for labelling regulation but also economic and ecological issues. Near infrared (NIRS) reflectance spectroscopy was employed to assess the origins of cuttlefish caught from five fishing FAO areas (Adriatic Sea, northeastern and eastern central Atlantic Oceans, and eastern Indian and western central Pacific Oceans). A total of 727 cuttlefishes of the family *Sepiidae* (*Sepia officinalis* and *Sepiella inermis*) were collected with a portable spectrophotometer (902–1680 nm) in a wholesale fish plant. NIR spectra were treated with standard normal variate, detrending, smoothing, and second derivative before performing chemometric approaches. The random forest feature selection procedure was executed to select the most significative wavelengths. The geographical origin classification models were constructed on the most informative bands, applying support vector machine (SVM) and K nearest neighbors algorithms (KNN). The SVM showed the best performance of geographical classification through the hold-out validation according to the overall accuracy (0.92), balanced accuracy (from 0.83 to 1.00), sensitivity (from 0.67 to 1.00), and specificity (from 0.88 to 1.00). Thus, being one of the first studies on cuttlefish traceability using NIRS, the results suggest that this represents a rapid, green, and non-destructive method to support on-site, practical inspection to authenticate geographical origin and to contrast fraudulent activities of cuttlefish mislabeled as local.

## 1. Introduction

Seafood authenticity represents a notable feature for governments, trades, and consumers. According to European Regulation (EU) n. 1379/2013 [1], fishery products must be labelled with the commercial designation, proper scientific name of the species, production method (caught or farmed), fishing gear (i.e., hook, trap, trawl), and catch or production area (FAO fishing area). Moreover, the name and the geographic origin of the fishery product allow to obtain information associable to some safety and regulatory aspects, in particular, to potential illegal fishing practice and to the presence of toxins, contaminant, or allergens that could represent a risk to human health and safety [2]. However, the complexity of the fishery supply chain and the loss of data or misinformation facilitate fraudulent activity in this sector, making seafood the second category of food most defrauded [3,4,5]. Indeed, among the non-conformity accounted for in the fishery sector, mislabeling was found to be the most recurrent commercial fraud (33%) [6]; the voluntary practice of labelling a lower value product as a higher value product is generally practiced for profit. In particular, the counterfeiting of the geographical origin, the sale of a thawed product as a fresh one, and specie substitution are the prominent issue in the fishery sector [7,8]. Such actions are made easier because it is difficult to monitor every phase of the whole supply chain [9]; hence, this lack results in the uncertain authenticity of the product on sale.

Cephalopods are considered one among the most valuable and abundant marine fishery products. Contrary to the intensive fishing of bone-fish stocks, the global biomass of cephalopods has increased; thus, greater fishing and trading of this product have been encouraged. Cephalopod world commerce has increased by 33% from 2010 to 2018 [10]. This sector represented the 7% of the total fishery trade [11], and in 2018, 2,223,854 tons were accounted for world exports of fresh, frozen, or chilled product [12]; in particular, the leading exporters were China (568,172 tons), Peru (209,162 tons), and India (187,472 tons) [12]. Cephalopods are widely appreciated by consumers both for the peculiar taste and for nutritional value; in particular, this seafood is mainly composed of water (80%), and it is considered a notable source of protein (16%) comparable to that provided by bone-fish and beef consumption [13,14]. However, cephalopods contain small amounts of fat (0.7–1.4%), similar to that observed in cod (0.7% [15]) but poorer than salmon (9% [16]) and beef (18% [17]). Moreover, this sea product is particularly rich in omega-3 (48% of total fatty acids), docosahexaenoic acid (30% of total fatty acids), and eicosapentaenoic acid (12% of total fatty acids [14]), and it is a good source of calcium, iron, and sodium [14]. Nutritional properties and geographic origin are among the most relevant aspects affecting consumer choice in food purchase [18]. Furthermore, geographical origin is one of the aspects currently most relevant to consumers due to the increasing awareness about the impacts of the purchasing choice of seafood on the marine environment [19]. Moreover, consumers generally prefer products from their own or a nearby nation with a short supply chain, which is perceived as safer and resulting in higher quality products [20].

The attribution of geographical origin in the seafood sector is generally performed in a laboratory through DNA- and protein-based techniques or isotopic measurements that require time, reagents, sample destruction, and trained personnel [21,22,23,24]. On the other hand, near-infrared spectroscopy (NIRS) is a technique widely employed to assess fishery product origin through a fast and easy evaluation [25,26,27]; in detail, this method does not require the sample destruction, the use of reagents, or personnel skilled to perform the analysis. Indeed, the award of origin through a rapid, green method applicable on-site represents an important strategy for food business operators, authorities, and regulators to meet internal traceability requirements and to implement and monitor the goods’ control in full chain traceability [19,28]. Studies that assess NIRS capability on cephalopods fraud have focused mainly on species substitution; few have aimed at the investigation of frozen-thawed product labelled as fresh [29,30]. However, although it has been used for other fish products [25,26,27], to our knowledge, no research has been conducted on the traceability of cephalopods through the NIRS technique. This study aimed to develop and validate through the use of machine-learning algorithms the classification model about geographic origin of individual cuttlefish (*Sepia officinalis* and *Sepiella inermis*) from five different FAO fishing areas.

## 2. Materials and Methods

### 2.1. Cuttlefish Sampling and Dataset

The study was carried out in an Italian wholesale fish plant located in Chioggia (Venezia, Italy). The sampling was conducted over 7 months (from November 2019 to July 2020), considering a total of 727 individual cuttlefish collected from 49 commercial batches. The dataset considered was composed by fresh (*n* = 221) and frozen-thawed cuttlefishes (*n* = 506) with different sizes (0.1–3.0 kg), originating from five catching areas and collected during four catching seasons (autumn, winter, spring, and summer). The detailed specification of all cuttlefish sampled, including the varieties, is presented in Table 1. Further specific information are reported in supplementary material (Table S1).

### 2.2. NIRS Data Collection

The NIRS data acquisition were carried out using a portable NIR spectrophotometer (PoliSPECNIR, ITPhotonics, Breganze, Italy) that operated in reflectance mode from 900 to 1680 nm with a resolution of 2 nm. Spectra of each cuttlefish were collected on intact and refrigerated cuttlefish (0–2 °C) after the usual company procedures (skinning, degutting, storage on ice). NIR spectral data measurements were performed through a round scanning window (3.2 cm^2^) placed in direct contact with the sample surface. Each spectrum was obtained by averaging of 5 s of data acquisition at a 10-msec integration time. Spectral data were registered in reflectance (R) units and converted to absorbance units as log (1/R) using poliDATA 3.0.1 software (ITPhotonics, Breganze, Italy).

### 2.3. Spectral Data Analysis

Data analysis was carried out by using R software, version 3.2.5 (R Core Team, 2016). Before any statistical analysis, spectra were pre-treated with standard normal variate (SNV), detrending [31], smoothing, and the second derivative to improve the spectral properties and remove the multiplicative interference of scattering [26,29,32].

A random forest (RF) feature selection procedure based on the Boruta algorithm (Boruta package, Comprehensive R Archive Network, R Development Core Team, 2010; [33]) with a wrapper approach was applied to select the most informative wavelengths and to remove unrelated and noisy data [26,34]. After feature selection and using the createDataPartition function of caret package of R [35], the whole dataset was split into a training set to perform the discrimination models and into a testing set to assess and validate the model developed. In particular, the training set was composed of 70% of the samples (*n* = 511), and the testing set consisted of 30% of the samples (*n* = 216). The model was validated through the hold-out validation in which the dataset was split again into the training set (70%) and used for the repeated cross-validation (setting number = 10 and repeats = 5) and the testing set (30%), composed of samples selected maintaining the proportionality among areas.

A principal component analysis (PCA) as an unsupervised method was performed to visualize the data distribution. Whereas, as supervised models, support vector machine (SVM) and K nearest neighbors (KNN) were used to investigate the NIR classification capability. The SVM was modelled by the use of the caret package through both the SVM-Linear and SVM-Radial kernels and applied to the training dataset. The C-value (Cost) in the Linear classifier and the radial basis function sigma were customized, adopting a grid search. The KNN was used as a classification method whose principle was to predict a class for a given test observed by attributing the class of the KNN observed sample. After training the models, the predict method was applied to obtain results in testing and validation. The model performance was evaluated through a confusion matrix, and the quality of prediction was assess by the accuracy, sensitivity, specificity, and precision metrics [36].

## 3. Results and Discussion

Nowadays, due to the increment of frauds identified in fishery sector, a strategic plan is required to detect and prevent seafood counterfeiting or mislabeling in short time due to the perishable nature of the product. Indeed, governments and food industry must take up actions against to this issue; however, in support of this claim, NIRS represent a valid approach widely applied in food control to meet the need for a rapid feedback in food authenticity. Thus, in the present study, NIRS was applied to collect spectral data and to discriminate cuttlefishes according to the origin of FAO fishing area using a total of 727 individual samples.

### 3.1. Feature Selection and Spectra Patterns

**Figure 1:**
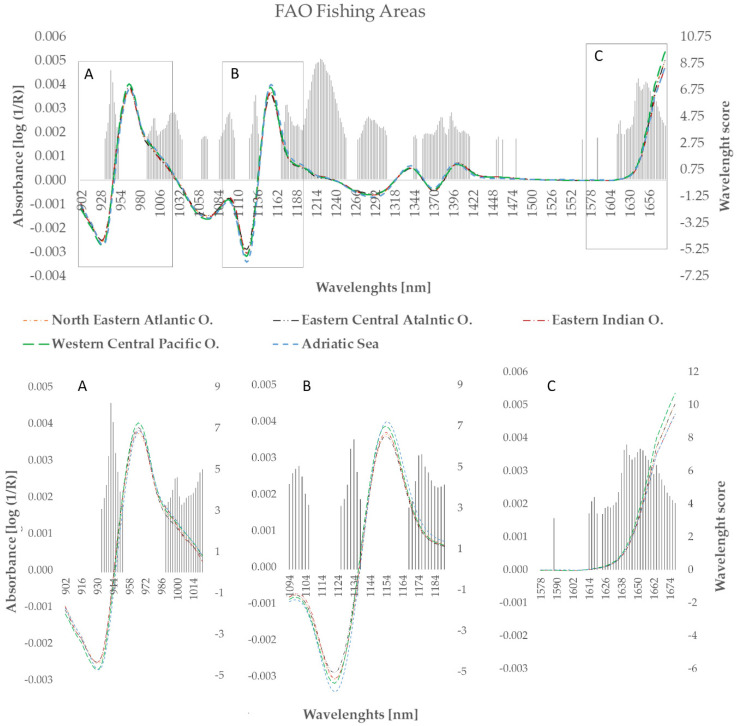
The mean NIR absorbance curves after standard normal variate, detrending, smoothing and the second derivative treatment and the most informative wavelengths selected by random forest (in grey) for predicting origin of FAO fishing area in cuttlefish. Magnification of spectra ranges where the differences in absorbance were detected (**A**) (902–1022 nm), (**B**) (1094–1190 nm), and (**C**) (1578–1678 nm).

Spectra samples was collected from 902 to 1680 nm in reflectance mode. However, among the 389 investigated wavelengths, only RF 194 resulted in significant results regarding the geographic origins that were considered for the further analyses; the most informative bands are related to the overtone and combination of some molecular chemical bonds, such as OH, CH, and NH [37,38]. The most prominent bands were observed as follows: around 934–952 nm (CH group, methylene, and hydrocarbons); around 990–1036 nm (nonbonded carboxylic acid hydroxyl, OH phenols, and NH amine); 1062–1070 nm (OH combination bands; alcohol or water); 1086–1106 nm (CH); 1126–1138 nm (CH, aromatic hydrocarbon); 1168–1254 nm and 1356–1418 nm (CH, hydrocarbons aliphatic); 1454–1456 nm (OH bond, water); 1462, 1480, and 1568 nm (NH bonds; amide, protein); 1588 nm (OH bond, alcohols or water); and 1614–1678 nm (CH and NH, alkenes and ketones). In particular, the most significant bands are mainly associated to methylene group of fat; in detail, absorption band around 930 nm and 1220 nm areas were related to the third and second overtone of CH stretch related to lipids, respectively [37,39]; in the present study, the resulting bands were characterized by the highest score (Figure 1; scores ranged between 3.0–8.2 and 6.0–9.0, respectively). Likewise, the area around the 1660 nm resulted in a compelling contribution to cuttlefish classification; indeed, also this area of the spectrum is ascribed to fat and fatty acids contents [26]. In particular, studies reported that fatty acids variability in seafood composition is affected mainly by fishing area since it is highly influenced by environmental and geographical aspects as water quality (i.e., temperature, salinity) and food source [40,41,42].

Figure 1 depicts the mean spectrum after pre-treatments according to the five geographic origins; in general, similar trends were observed among the mean NIR spectra of five FAO fishing area. However, slight differences were observed at 932, 966, 1122, 1154, and 1678 nm that are probably due to the differences among the spectra related to hydrocarbons, alkyl alcohols, aromatic amine, and ketones [38]; in particular, the mean spectrum of cuttlefishes caught in the Adriatic Sea is characterized by highest absorbance; on the other hand, the lowest absorbance values were observed in eastern central (EC) Atlantic Ocean.

### 3.2. Principal Component Analysis

**Figure 2:**
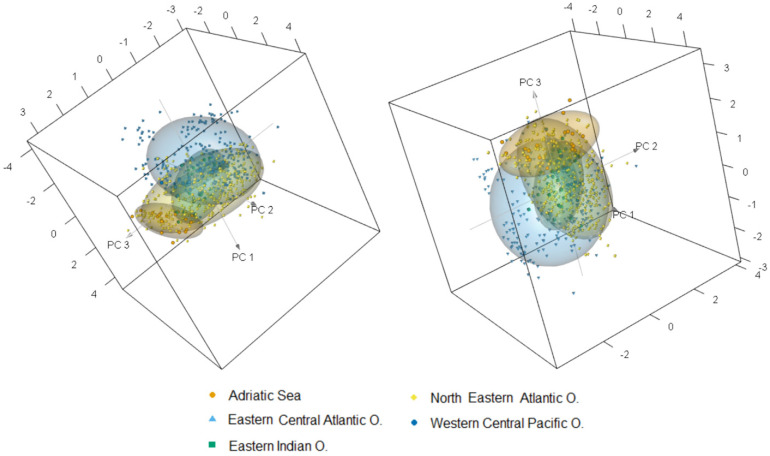
Principal component score plot for PC1, PC2, and PC3 of cuttlefishes originating from five FAO fishing areas. Ellipses represent the 75% confidence interval.

Subsequently to the feature wavelengths selection, the PCA and classification models have been designed using only the most informative wavelengths of pre-treated data to discard irrelevant attributes. The PCA is performed as an unsupervised approach to visualize the graphical data distribution, offering a simple method to detect a potential clustering of samples according to the caught fishing area. The cumulative contribution of the first three PCs accounts for 76% of the total variance of NIRS data; in particular, PC1 explains 39% of the total variance, whereas PC2 and PC3, 21% and 16%, respectively. Figure 2 shows the score of the first three PCs, showing valuable clustering among groups of different geographic origins; in detail, a noticeable overlap of the data distribution among the five classes was observed among the contiguous areas, as between the (northeastern) NE and EC Atlantic Oceans and between the eastern (E.) Indian and western central (WC) Pacific Oceans. This is probably due to the high collinearity in the variables among classes or from the large spread of sample data points. On the other hand, samples caught in the Adriatic Sea resulted in a distinct separation from the other areas; however, a slight overlapping with NE Atlantic Ocean was observed probably due to the highest spread of Atlantic data points.

### 3.3. Machine-Learning Analyses

The classification of foodstuff combining NIRS with chemometric approaches has already been considered as valid method to discriminate the origin of vegetable products, such as asparagus [43] and rice [44], and in fishery product, as observed in sea cucumber [25], sea bass [26], tilapia [27], and anchovies [41]. In detail, in the present study, the capability of the NIRS to discriminate cuttlefish according to the FAO fishing area was assessed comparing two among the most common chemometric techniques used in food authentication and adulteration [45], i.e., SVM and KNN models. In particular, SVM and KNN algorithms were performed as supervised methods and compared to each other to evaluate the best classifier model. However, between those two discriminant approaches, the SVM model demonstrated the best performance of classification even with unbalanced data among classes, as reported in the study of Farquad and Bose [46]. Models’ performance were evaluated based on overall accuracy, balanced accuracies, and on the ability to classify sample origin correctly as belonging (sensitivity) or not belonging (specificity) to a specific origin class [47,48]. In general, the KNN performance was poorer than that observed for SVM; in particular, KNN showed an overall accuracy of 0.84, and the greatest specific balanced accuracy was 0.88. Specificity ranged between 0.80 and 1.00, whereas sensitivity varied from 0.20 to 0.93. Instead, the SVM model yielded the greatest performance of classification; thus, SVM results are reported in Table 2 and discussed more in detail.

The SVM gave an overall accuracy of 0.92, showing the greatest number of samples assigned correctly to their own class; on the other hand, 8% (*n* = 18) of samples were incorrectly classified. Notably, the balanced accuracy reported for the NE Atlantic and EC Atlantic Oceans were 0.92 and 0.93, respectively. In particular, the 12% (*n* = 10) of samples from EC Atlantic Ocean were assigned to NE Atlantic one; thus, a spectra of samples from EC Atlantic Ocean could be characterized by traits similar to those of the NE Atlantic Ocean. However, in NE Atlantic Ocean class, we observed the lowest misclassification occurrence (3.6%, *n* = 4), with samples erroneously assigned to the EC Atlantic Ocean (*n* = 2) and to E. Indian and WC Pacific Oceans (1 sample per both). Among the balanced accuracy results, however, the lowest value (0.83) was attributed to WC Pacific Ocean, which showed the lowest sensitivity (0.67) in which 33% (*n* = 3) of samples were misclassified as belonging to the NE Atlantic Ocean (22%; *n* = 2) and to EC Atlantic Ocean (11%; *n* = 1). Moreover, the balanced accuracy reported for Adriatic Sea was 0.90, showing that the 20% of samples (*n* = 1) were considered to belong to the NE Atlantic Ocean. However, the best performance of the SVM algorithm was represented by the greatest balanced accuracy, sensitivity, and specificity of 1.00 observed for the E. Indian Ocean class in which samples were totally discriminated from the other groups. In detail, the results observed on *Sepiella inermis* show that samples (*n* = 8) from E. Indian Ocean were completely separated from cuttlefish of other origins regardless to the species, size, and physical status; indeed, the highest and whole accuracy of sample classification from E. Indian Ocean could be attributed to the significantly different water environments and food sources in respect to those of the *Sepiella inermis* from WC Pacific Ocean.

The findings observed in this study are in accordance with those observed in literature considering other sea products. In detail, the overall accuracy of the present study (0.92) was greater than one (0.89) reported in the research about the geographical classification of European sea bass (*Dicentrarchus labrax*) proposed by Ghidini et al. [26]. The discrepancy with the Ghidini et al. [26] study could be related to the small area considered (west, central and eastern Mediterranean Sea), to the low sample size (*n* = 144), or to the different model of classification considered (OPLS-DA) in their study. On the other hand, the study focused on the traceability of Chinese tilapia from four geographical origins, conducted by Liu et al. [27], showed the highest ratio (0.85) of samples correctly classified, lower than the present study (1.00). In detail, differences could be related to the lower sample variability due to the small sampling period (October to December), small size of sample (*n* = 208), and different chemometric approach (SIMCA) considered in their study [27]. On the other hand, the research performed by Guo et al. [25] that discriminated 189 sea cucumbers (*Apostichopus japonicus*) according to the geographic origin from nine different origins of Chinese sea reported a higher value of classification rates (1.00) for all four classes considered; this is probably due to the lower variability given by the smaller size of sample (*n* = 45) employed in the validation test than in the present study. Moreover, compering the results reported in this study to the ones in the previous study that were considered standard techniques to discriminate seafood according to the geographical origin, it is important to consider that NIRS technique is a fast, non-consuming, green, and easy approach. Indeed, the common techniques are generally performed on a small-sized sample and require a long analytical phase performed by skilled personnel. In particular, the findings observed in the research conducted by Varrà et al. [49] resulted in higher accuracy than the present study; in detail, Varrà et al. [49] reported the whole sensitivity, specificity, and accuracy (1.00) in the classification of cuttlefishes according to the geographic origin through the quantification of the elemental composition defined using a laboratory instrument of 68 samples. However, although the findings observed in this study were slightly lower than those of Varrà et al. [49], the importance of the present study is attributable to a great variability of data collected (*n* = 727), evidencing the effectiveness of classification according to the geographical origin in employing a portable and fast tool in a real environment, which is a real stage of the cuttlefish supply chain. Moreover, for other approaches generally employed to discriminate according to the geographical origin, such as DNA or isotopic techniques, these are time consuming and expensive. Furthermore, the isotopic analysis shows limitation in accuracy results being affected by environmental effects, such as diet, season, and salinity [19]; in fact, the isotopic technique is generally supported by the elemental technique to improve the accuracy of food traceability [19]. Such achievements confirm the applicability of NIRS technology as a portable, independent, and valid tool suitable during on-site inspection in every phase of the cuttlefish supply chain to control and prevent frauds of origin mislabeling.

## 4. Conclusions

This study demonstrates the feasibility of NIRS in classification according to geographical origins, achieving an accuracy of 0.92 when using a large sample dataset (*n* = 727) collected from five FAO fishing areas. Spectral data collection was performed in a fishing plant to replicate the real conditions of control inspection executed in a part of the supply chain. The cuttlefish discrimination according to the caught area is achievable though the environmental and geographical aspects that affect the chemical composition of samples. This result suggests that the employment of a portable NIRS instrument is a user-friendly, fast, suitable, and independent analytical approach for supporting the regulatory inspection that awards the geographic origin on-site for perishable products. NIRS approach represents an important strategy for food business operator, authorities, and regulators to meet internal traceability requirements and to implement and monitor the control of goods in full chain traceability.

## Figures and Tables

**Table 1:** Geographic origin, correspondent FAO fishing area, and description of cuttlefish species included in the study.

Geographic Origin	FAO Fishing Area	Species	Samples
Northeastern Atlantic Ocean	27	*Sepia officinalis*	371
Eastern central Atlantic Ocean	34	*Sepia officinalis*	279
Eastern Indian Ocean	57	*Sepiella inermis*	28
Western central Pacific Ocean	71	*Sepiella inermis*	30
Adriatic Sea	37.2.1	*Sepia officinalis*	19

**Table 2:** Performance of classification of support vector machine model to discriminate cuttlefish according to the origin in hold-out validation.

	Reference Classes				
	Northeastern Atlantic O.	Eastern Central Atlantic O.	Adriatic Sea	Eastern Indian O.	Western Central Pacific O.
**Predicted Classes**					
Northeastern Atlantic O.	107	10	1	0	2
Eastern Central Atlantic O.	2	73	0	0	1
Adriatic Sea	0	0	4	0	0
Eastern Indian O.	1	0	0	8	0
Western Central Pacific O.	1	0	0	0	6
Sensitivity	0.96	0.88	0.80	1.00	0.67
Specificity	0.88	0.98	1.00	1.00	1.00
Balanced Accuracy	0.92	0.93	0.90	1.00	0.83

## Data Availability

Not applicable.

## Supplementary Materials

The following are available online at https://www.mdpi.com/article/10.3390/foods10081678/s1, Table S1. Descriptive information of cuttlefish sampling data considered in the study.
