# Supplementary material for: Fast and Green Method to Control Frauds of Geographical Origin in Traded Cuttlefish Using a Portable Infrared Reflective Instrument
Source: Foods. 2021 Jul 21;10(8):1678. doi: 10.3390/foods10081678 (PMC8391955; doi:10.3390/foods10081678)
Supplement: Supplementary file 1 [file foods-10-01678-s001.zip › foods-1292498-supplementary.pdf]

Table S1. Descriptive information of cuttlefish sampling data considered in the study.

Date of sampling	Batch	N. of samples	Physical status	Species	Geographic origin
29.11.2019	273/19	10	Fresh	*S. officinalis*	27
29.11.2019	802/19	10	Frozen-Thawed	*S. officinalis*	34
06.12.2019	293/19	10	Fresh	*S. officinalis*	27
06.12.2019	824/19	10	Frozen-Thawed	*S. officinalis*	34
07.01.2020	03/20	18	Fresh	*S. officinalis*	27
09.01.2020	05/20	10	Fresh	*S. officinalis*	27
09.01.2020	580/19	10	Frozen-Thawed	*S. officinalis*	27
14.01.2020	863/19	30	Frozen-Thawed	*S. officinalis*	27
16.01.2020	33/20	15	Fresh	*S. officinalis*	27
17.01.2020	33/20	15	Frozen-Thawed	*S. officinalis*	34
20.01.2020	53/20	16	Fresh	*S. officinalis*	27
20.01.2020	41/20	15	Frozen-Thawed	*S. officinalis*	34
23.01.2020	38/20	15	Frozen-Thawed	*S. inermis*	71
27.01.2020	50/20	15	Frozen-Thawed	*S. officinalis*	34
30.01.2020	66/20	20	Fresh	*S. officinalis*	27
06.02.2020	77/20	16	Fresh	*S. officinalis*	27
06.02.2020	76/20	10	Fresh	*S. officinalis*	37.2.1
13.02.2020	90/20	21	Fresh	*S. officinalis*	27
13.02.2020	91/20	17	Fresh	*S. officinalis*	27
18.02.2020	99/20	10	Fresh	*S. officinalis*	27
21.02.2020	102/19	12	Fresh	*S. officinalis*	27
21.02.2020	93/20	10	Frozen-Thawed	*S. officinalis*	34
03.03.2020	113/20	12	Fresh	*S. officinalis*	27
03.03.2020	116/02	15	Fresh	*S. officinalis*	27
03.03.2020	698/19	19	Frozen-Thawed	*S. officinalis*	27
05.03.2020	107/20	15	Frozen-Thawed	*S. officinalis*	27
10.03.2020	699/19	20	Frozen-Thawed	*S. officinalis*	34
06.05.2020	169/20	7	Frozen-Thawed	*S. officinalis*	34
06.05.2020	161/20	19	Frozen-Thawed	*S. inermis*	57
11.05.2020	178/20	22	Frozen-Thawed	*S. officinalis*	27
12.05.2020	179/20	14	Frozen-Thawed	*S. officinalis*	27
19.05.2020	184/20	14	Frozen-Thawed	*S. officinalis*	34
23.05.2020	152/20	9	Fresh	*S. officinalis*	37.2.1
09.06.2020	201/20	9	Frozen-Thawed	*S. inermis*	57
16.06.2020	207/20	9	Frozen-Thawed	*S. officinalis*	34
23.06.2020	224/20	14	Frozen-Thawed	*S. officinalis*	34
30.06.2020	236/20	21	Frozen-Thawed	*S. officinalis*	27
30.06.2020	245/20	9	Frozen-Thawed	*S. officinalis*	34
07.07.2020	231/20	15	Frozen-Thawed	*S. officinalis*	34
08.07.2020	333/20	18	Frozen-Thawed	*S. officinalis*	27
08.07.2020	232/20	15	Frozen-Thawed	*S. officinalis*	34
08.07.2020	232/20	20	Frozen-Thawed	*S. officinalis*	34
15.07.2020	267/20	9	Frozen-Thawed	*S. officinalis*	34
15.07.2020	271/20	11	Frozen-Thawed	*S. officinalis*	34
20.07.2020	277/20	31	Frozen-Thawed	*S. officinalis*	34
20.07.2020	195/20	15	Frozen-Thawed	*S. inermis*	71
21.07.2020	252/20	20	Frozen-Thawed	*S. officinalis*	34
29.07.2020	334/20	20	Frozen-Thawed	*S. officinalis*	27
29.07.2020	297/20	10	Frozen-Thawed	*S. officinalis*	34

Area FAO: 27, North Eastern Atlantic Ocean; 34, Eastern Central Atlantic Ocean; 57, Eastern Indian Ocean; 71, Western Central Pacific Ocean; 37.2.1, Adriatic Sea.
